# A new therapy (MP29-02*) effectively treats patients with seasonal allergic rhinitis who suffer most from the bothersome nasal symptom of congestion

**DOI:** 10.1186/2045-7022-3-S2-P39

**Published:** 2013-07-16

**Authors:** Claus Bachert, David Price, Glenis Scadding, Wytske Fokkens, Peter Hellings, Ullrich Munzel, Jean Bousquet

**Affiliations:** 1 Ghent University Hospital, Dept of Oto-rhinolaryngology, Ghent, Belgium; 2 University of Aberdeen, Dept of General Practice & Primary Care, Abderdeen, UK; 3 The Royal National Throat, Nose and Ear Hospital, London, UK; 4 Academic Medical Center, Dept of Otorhinolaryngology, Amsterdam, the Netherlands; 5 University Hospitals Leuven, Dept of Otorhinolaryngology, Head and Neck Surgery, Leuven, Belgium; 6 Meda Pharma, Biostatistics & Market Access, Bad Homburg, Germany; 7 Hopital Arnaud de Villeneuve University Hospital, Montpellier, France

## Background

In clinical practice, allergic rhinitis (AR) patients frequently present with a predominant or particularly bothersome symptom, most frequently nasal congestion.

## Objective

To assess the efficacy of MP29-02* (a novel intranasal formulation of azelastine hydrochloride [AZE] and fluticasone propionate [FP]) in patients with seasonal AR (SAR) suffering predominantly from nasal congestion, compared to commercially available AZE or FP nasal sprays and placebo.

## Methods

610 patients (≥12 years old) with moderate-to-severe SAR were randomized into a double-blind, placebo-controlled, 14-day, parallel-group trial to MP29-02*, AZE or FP nasal sprays and placebo (all given as 1 spray/nostril bid; total daily dose: 548µg AZE, 200µg FP]. Patients were defined as 'nasal congestion predominants' if their maximum symptom score at baseline was the nasal congestion score (n=368). Both reflective total nasal symptom score (rTNSS; max score =24) and nasal congestion symptom score (max score =6) reduction were assessed in these patients to show effect on their overall nasal symptom burden, as well as specific relief from nasal congestion.

## Results

MP29-02* induced the greatest reduction in rTNSS in patients complaining of nasal congestion (-5.64), compared to -3.93 for FP (Diff -1.71; 95% CI -3.00, -0.43; p=0.0093), -3.28 for AZE (Diff -2.36; 95% CI -3.51, -1.21; p<0.0001) and -2.63 for placebo (Diff -3.01; 95% CI -4.14, -1.88; p<0.0001), corresponding to a relative treatment difference of 57% to FP and 79% to AZE. These nasal congestion-predominant patients treated with MP29-02* also experienced a significantly greater reduction in their nasal congestion score; -1.41 vs -0.90 for FP (Diff: -0.51; 95% CI -0.83, -0.19; p=0.0018), -0.83 for AZE (Diff: -0.58; 95% CI -0.88, -0.29; p=0.0001) and -0.69 for placebo (Diff -0.72; 95% CI -1.02, -0.42; p<0.0001), with a relative treatment difference of 71% to FP and 81% to AZE. Neither AZE nor FP significantly differed from placebo in terms of nasal congestion reduction in these patients.

## Conclusion

Unlike currently available first line therapy, MP29-02* effectively reduced nasal congestion and the overall nasal symptom burden of patients suffering predominantly from nasal congestion. This indicates that for nasal congestion predominant patients a decongestant might not be required prior to MP29-02* administration, and further supports the position of MP29-02*as the drug of choice for the treatment of AR.

*Dymista

